# Hybrid solar photovoltaic conversion and water desalination via quad-band fano-resonant optical coatings and superwicking cooling

**DOI:** 10.1038/s41377-025-01796-z

**Published:** 2025-04-17

**Authors:** Ran Wei, Tianshu Xu, Mingjiang Ma, Mohamed Elkabbash, Chunlei Guo

**Affiliations:** 1 The Institute of Optics, University of Rochester, Rochester, NY USA; 2 Wyant College of Optical Sciences, University of Arizona, Tucson, AZ USA

**Keywords:** Laser material processing, Solar energy and photovoltaic technology

## Abstract

Hybrid Photovoltaic/Thermal (HPT) systems simultaneously convert solar energy into electrical power and thermal energy. These systems are attractive as they enable the thermal management of PV cells to maintain optimal operating temperatures and maximize the overall solar energy conversion. Despite their advantages, HPT systems have been limited to storing solar energy in the form of heat or simple water/space heating, thus restricting the broader application scope of HPT systems, particularly in regions with abundant solar energy. Here, we introduce a device that expands the scope of HPT applications by realizing a hybrid PV/ water desalination system, achieved through the integration of a Fano-resonant optical coating (FROC) onto a silicon substrate, which is turned superwicking via femtosecond laser surface patterning. This configuration allows a single-junction amorphous silicon solar cell to operate under higher solar concentrations with much less heat conversion, achieving a temperature reduction of 101 °C and an efficiency improvement of 335.7% compared to a standalone photovoltaic system under the solar concentration of 5. At the same time, the interfacial water desalination achieves a 2 ${kg}{m}^{-2}{h}^{-1}$ high evaporation rate. Over a 12-hour cycle, our HPT system showed a consistent performance, demonstrating a combined solar conversion efficiency of 79.6%. The demonstrated superwicking-FROC will pave the way for widespread adoption of HPT systems particularly in sunny coastal regions.

## Abstract (web summary)

Schematics of the superwicking-FROC solar hybrid photovoltaic/thermal system. This system provides simultaneous high efficiency electricity generation and on-site water desalination.

## Graphical abstract

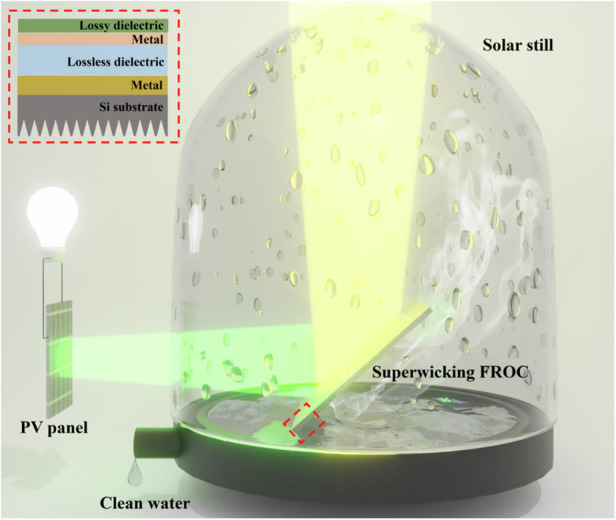

## Introduction

The escalating global energy demand, driven by increasing populations and industrialization, has led to a surge in greenhouse gas emissions and an urgent need for clean and sustainable energy solutions, chief among them is solar power. The deployment of solar panels, engineered to capture and convert sunlight into electricity through photovoltaic (PV) systems^1,2^, has garnered immense popularity across residential, commercial, and industrial sectors due to its scalability, cost-efficiency, and minimal environmental impact.

**Fig. 1:**
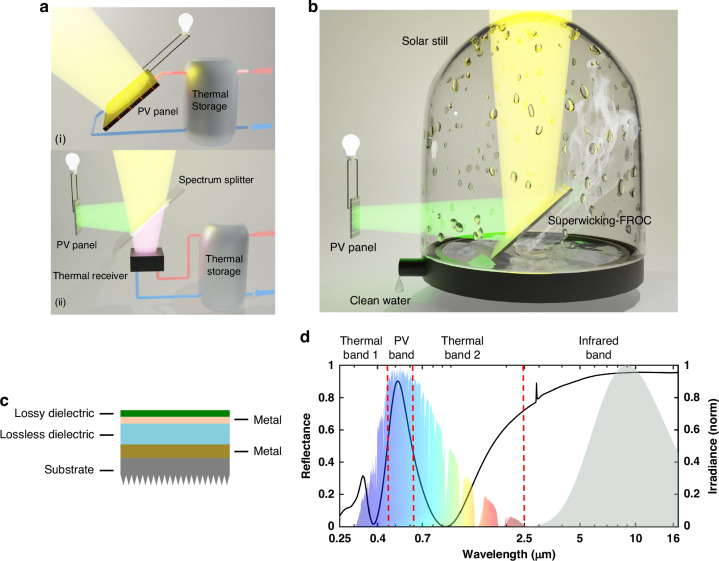
**Schematics of the proposed superwicking-FROC HPT system. a** Schematics of (i) a thermally coupled HPT system and (ii) a thermally decoupled HPT system, where the thermal energy is mostly used to heat up the water. **b** Schematics of the proposed superwicking-FROC HPT system, which provides simultaneous high-efficiency electricity generation and on-site water desalination. **c** Schematics of a four-layer FROC deposited on a Si substrate with backside turned superwicking, with a distinctive (**d**) quad-band spectral response containing one reflective narrow PV band, two thermal bands for effective absorption of solar energy, and one infrared band to minimize thermal losses

However, in a standalone solar PV system, only photon energy around the bandgap of the PV cell can be converted into electricity, while the remaining photon energy will be absorbed and converted to heat within the PV cell. The heat conversion limits the solar conversion maximum theoretical efficiency. Moreover, the generated heat reduces the PV cell efficiency due to increased carrier recombination rates and decrease in the cell’s bandgap^3^. Meanwhile, the heat reduces the PV cell’s lifetime, with the aging rate nearly doubles with every 10 °C increase in temperature^4^. Solar hybrid photovoltaic/thermal (HPT) systems are a potential solution to both the reduced solar conversion efficiency and thermal management of PV cells^5–7^. HPT systems can be divided into two categories: thermally coupled HPT^7^ and thermally decoupled HPT system^8^. The former employs a cooling package adhered to the back of the PV cell for effective temperature control as well as fluid heating with the waste heat (Fig. 1(a;i)). Nevertheless, the cooling capabilities are often inadequate under concentrated solar illumination^9^. Thermally decoupled HPT system, on the other hand, separates incident solar energy into PV and thermal bands with spectrum splitter and directs the split solar to the respective PV and thermal module (Fig. 1(a;ii))^10^, enabling high solar concentration with better temperature control. In both cases, the captured thermal energy is mostly employed to heat up the circulating fluid for versatile residential or industrial purposes^11–14^. Consequently, the HPT system optimally distributes available solar energy across the respective PV and thermal module, ensuring a more resource-efficient utilization.

To realize better thermal management in thermally decoupled HPT systems, quad-band spectrum-splitting filter were proposed and demonstrated^15,16^. In a quad-band filter, the entire solar spectrum is separated into a PV band, two thermal bands, and an infrared (IR) band using a dielectric Bragg reflector (DBR). However, DBR-based HPT systems require additional thermal receivers and face integration complexities due to substrate-dependent spectral responses. Moreover, DBR mirrors are typically tens of microns thick, making them relatively costly to produce.

Meanwhile, the imbalance between the increasing demand for clean water and its diminishing availability, mainly due to population growth and climate change, has become a critical global challenge^17^. Conventional approach to obtaining clean water largely relies on reverse osmosis^18^ but the process itself is energy intensive with large carbon footprints^19,20^. Solar-thermal interfacial desalination, however, represents an environmentally feasible and economically viable solution to address the global shortage of clean water^21–23^. A highly integrated interfacial evaporator utilizing single-step femtosecond laser writing has been developed recently^24^, where a flexible superwicking and solar absorbing metal foil functions as the key component for simultaneous solar absorption and water evaporation. While prior research studied water desalination with thermally coupled HPT systems^25–28^, the thermal energy was limited to pre-heat the saline water driven by the water pump to enhance the throughput of subsequent off-site desalination processes, such as reverse osmosis, thermal methods, or solar stills. Direct on-site water desalination using the thermal module in the thermally decoupled HPT system, nevertheless, remains unexplored.

Here, we introduce a compact thermally decoupled HPT system consisting of a thermal module designed for direct water desalination and a PV module for power generation (Fig. 1(b)). This HPT system consists of three components: (i) a PV cell, (ii) a dual-purpose spectrum splitter with one side being superwicking to conduct efficient water desalination^24^ and (iii) the other side separates the incident solar spectrum into four spectral bands with a Fano Resonant Optical Coating (FROC, Fig. 1(c))^29^. The FROC consists of only four-layer coatings with a total thickness being 1 order of magnitude thinner than a typical DBR mirror, which will roughly translate into a proportional cost saving of 10 times. We name this combined superwicking substrate and FROC as superwicking-FROC. The quad-band spectral response of FROC exhibits a weak angular dependence, which makes it function as a PV-band reflector over a wide range of incident angles and as an efficient thermal receiver to harness the thermal energy with minimal losses. The PV band reflector allows a single-junction solar cell to work under the concentrated solar light beyond the AM 1.5 G solar illumination with a proper working temperature, while the solar thermal energy is directly used for efficient on-site water desalination with the superwicking surface without the need for external pumps. Moreover, the continuous desalination also enables the superwicking-FROC to maintain an appropriate temperature such that the spectral response remains the same under high solar concentrations. Such a single component with combined optical filtering and thermal management performance has never been realized in the past until this work. Under the optimal solar concentration, the superwicking-FROC HPT system improves the photoelectric conversion efficiency by 335.7% compared to a traditional solar PV system operating under the same condition. This enhancement stems from FROC’s spectral design (Fig. 1(d)), significantly reducing the heat generation within the PV cell, which could lead to a twenty-fold increase in the PV cell lifetime. Similarly, we show that as the superwicking FROC desalinate water, its temperature is regulated, which maintains the mechanical and optical integrity of the thin film stack. The proposed system offers a scalable, cost-effective solution poised for widespread adoption, potentially transforming the renewable energy and water purification markets.

## Results

In this section, we present the findings and comprehensive discussions of our study. We will first go through the design of the FROC as a quad-band spectrum splitter, where the performance of the as-deposit FROC is examined. Next, the superwicking silicon (Si) realized through direct femtosecond (fs) laser writing is systematically studied and optimized for the best evaporation performance. Afterwards, the self-thermal-management functionality of the superwicking-FROC is carefully studied. Eventually, the performance of the superwicking-FROC HPT system is tested, where the output electricity as well as the temperature of the PV cell are carefully examined. Over a 12-hour cycle, the performance of the superwicking-FROC HPT system is tested for simultaneous electricity generation and water desalination.

### Fano-resonant optical coating (FROC) as a quad-band spectrum splitter

Fano resonance results from the coupling of a discrete localized state to a continuum state to create an asymmetric resonant line profile^30^, which was first discovered in atomic physics but has recently gained attention in nanophotonics^30,31^. While prior demonstrations relied on complex nanofabrication techniques^32–34^, a novel design has been recently proposed employing ultrathin film optical coatings, i.e., Fano resonant optical coatings (FROCs)^29^. The asymmetric resonant line profile of a FROC is realized by coupling a narrowband absorbing thin film structure, representing the discrete state, with a broadband absorbing thin film structure, representing the continuum state. As a result, the thin film stack exhibits a narrowband reflection with Fano line shape.

**Fig. 2:**
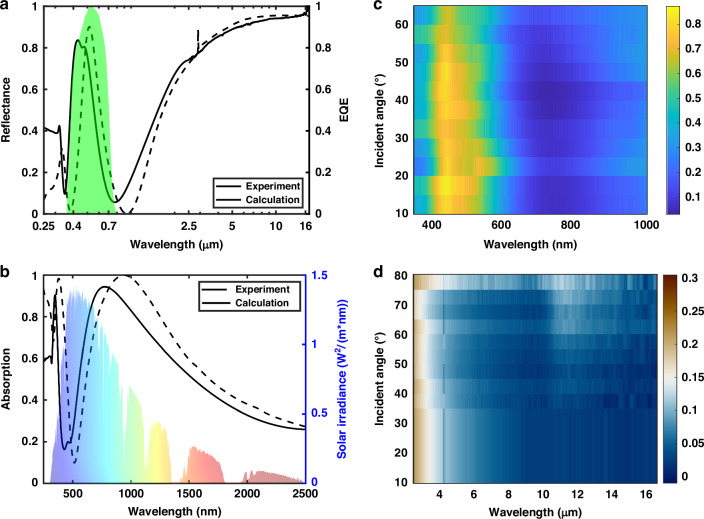
**Design and characterization of the quad-band FROC. a** Comparison of the reflectance over UV-vis-IR spectral range for the numerically calculated and as-deposit FROC, respectively. The shaded green area represents the external quantum efficiency of the amorphous silicon solar cell. **b** Absorption comparison over the solar spectral range for the numerically calculated and as-deposit FROC, respectively. **c** Experimentally measured angular reflectance in the visible range for FROC under p-polarized illumination. **d** Experimentally measured angular absorptance/emittance in the IR range for FROC

To function as an effective spectrum splitter, the spectral response of the FROC necessitates careful design: it should reflect a narrowband of light centered around the material’s bandgap (e.g., amorphous Si) while efficiently absorbing and storing the remaining solar energy as heat without significant thermal loss. To meet these criteria, a quad-band FROC with 4-layer thin film optical coating of Ge (9 nm) – Ni (9 nm) – TiO_2_ (73 nm) – Ag (100 nm) was designed, effectively splitting the spectrum into a PV band, two thermal bands, and an IR band. The PV band was designed to perfectly overlap with the peak of the external quantum efficiency of the PV cell (Fig. 2(a)). The first thermal band shows strong absorption in the UV region of the solar spectrum, while the other thermal band follows the solar spectrum with a low absorption tail, ensuring reduced emission across the IR band with suppressed radiation loss. Consequently, the quad-band FROC spectrum splitter ensures high-efficiency electricity generation with negligible temperature elevation while simultaneously enabling the efficient storage of excess solar energy as heat with minimal losses.

The solid black line in Fig. 2(a) presents the reflectance measured for the as-deposit FROC on a truncated 50 mm × 90 mm silicon (Si) wafer, which shows a slight blue-shift in the reflection peak of approximately 77 nm from our design (dotted black line). The two absorptive thermal bands shifted accordingly and had diminished peak intensities, as illustrated in the absorption measurement in Fig. 2(b). The measured spectral responses in Fig. 2(a) and (b) are complementary to each other since the FROC is opaque. The deviation in the spectral response for the as-deposit FROC arises from inaccuracies of the deposition system. Nevertheless, a reasonably favorable agreement is obtained between the theoretical calculations and the as-deposit FROC, where the four bands can be distinctively visualized.

Depending on its design, FROC can exhibit ultra-low iridescence^29^. We numerically calculate the angular reflectance of p-polarized and s-polarized light for the FROC across the solar spectral range, respectively, as illustrated in Fig. S1(a) and (b). Under both polarizations, the reflectance of FROC displays angular independence up to 80°. This ensures a consistent reflection of the same PV band towards the PV cell, enabling solar trackability and concentrated solar for enhanced photoelectric conversion. The measured angular reflectance of the as-deposit FROC within the solar spectral range (Fig. 2(c) and Fig. S1(c) for s-polarized light) and the IR range (Fig. 2(d)) demonstrates good agreement with the simulations and experimentally validates the FROC’s low iridescence.

### Superwicking silicon surface

**Fig. 3:**
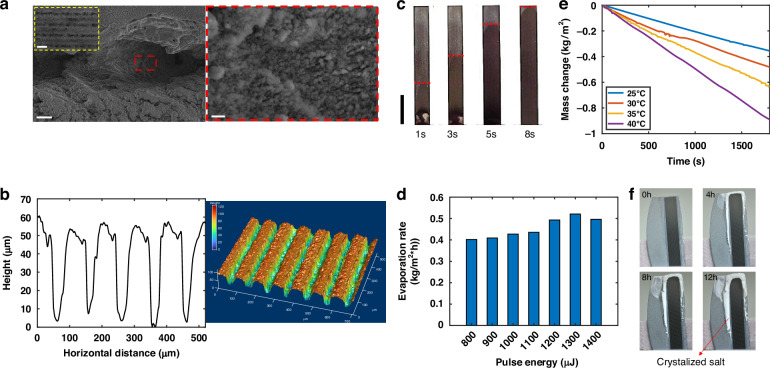
**Characterization and desalination performance of superwicking Si. a** SEM images of the microchannels written by fs laser and zoom-in view of the surface micro- and nano- structures. The scale bars are 80 μm, 10 μm, and 400 nm for the three magnifications, respectively. **b** Profile of the microchannels and the corresponding 3D view. **c** Real-time capturing of waterfront positions when the bottom of the superwicking Si touches water. The scale bar is 10 mm. **d** Evaporation rates for superwicking Si fabricated under different pulse energies. **e** Water mass change curves for the superwicking Si measured under different ambient temperatures. **f** Photographs taken at different time of the superwicking Si desalinating 3.5% wt saline water at an ambient temperature of 40 °C, where the active evaporation area (i.e., black region) remains free from salt crystallization

The unique spectral response enables the storage of excess solar energy as heat within the FROC, which can support highly efficient on-site solar-thermal interfacial desalination to obtain clean water. This is made possible by the superwicking Si surface through a single-step fs laser treatment^35,36^. Figure 3(a) presents the scanning electron microscopy (SEM) images of the laser-treated Si surface, where the microchannels are covered by ample micro- and nano- structures. Figure 3(b) shows the profile of the microchannels produced at a laser pulse energy of 1300 $\mu J$, characterized by an average depth of 50 $\mu m$ and an average full width at half maximum (FWHM) width of 31 $\mu m$. Our evaporation tests showed this microchannel structure gives the best evaporation performance, as to be discussed in detail later. The deep and narrow microchannels and the enhanced surface roughness ensure exceptional capillary effect^24,37^ and concurrently unprecedented superwicking property, which is confirmed by the contact angle measurement (see Fig. S2) and the water transport measurement (Figs. 3(c) and S3(a)). Upon contacting the superwicking Si, the water quickly spreads uphill along the capillaries and forms a thin water layer covering the entire surface, allowing high-efficiency water desalination.

In order to have a superwicking Si surface with the optimal evaporation performance, seven superwicking Si samples with equal sizes (5 mm $\times$ 30 mm) are prepared following different pulse energies spanning from 800 $\mu J$ to 1400 $\mu J$ with an interval of 100 $\mu J$ through a Galvanometer scanner (see Methods). Evaporation measurements are conducted by inserting each superwicking Si sample into a water reservoir with the bottom just touching the water and continuously monitoring the mass reduction in half an hour in evaporating the distilled water (see Methods for more measurement details). Evaporation rates for all samples are approximated through linear fitting from the mass change curves (Fig. S3(b)) and are summarized in Fig. 3(d). Maximum evaporation rate of 0.52 ${kg}{m}^{-2}{h}^{-1}$ is realized at a pulse energy of 1300 $\mu J$ under room temperature (20 °C, relative humidity 40%).

Furthermore, we constructed an environmental chamber to evaluate the evaporation performance of the superwicking Si with distilled water under elevated temperatures. Figure 3(e) depicts the corresponding mass change curves observed over a half-hour measurement period at four distinct ambient temperatures (25 °C, 30 °C, 35 °C, and 40 °C). The evaporation rate exhibits a remarkable enhancement as the temperature rises. Specifically, at an ambient temperature of 40 °C, the evaporation rate reaches to 1.78 ${kg}{m}^{-2}{h}^{-1}$, representing more than threefold increase compared to the evaporation rate observed at room temperature in Fig. 3(e). The increment in the evaporation rate of the superwicking Si with elevated temperature is verified by monitoring the surface temperature and the relative humidity in the environmental chamber, which can be found in Supplementary Note 2.

Additionally, superwicking Si surface exhibits an intriguing self-clean property when evaporating saline water, where the active evaporating area remains free from salt accumulation after 12 h continuous operation in desalinating 3.5% wt saline water (Figs. 3(f) and S4), avoiding clogging issue encountered by conventional interfacial evaporators with performance degradation^38^. We believe that this self-clean salt rejecting phenomenon is a combined result of coffee-ring^39^ and the salt creeping effect^40^, where the former one induces initial crystallization of salt at the edge of the active area and the latter precipitates the salt crystallization far from the evaporating salt solution boundary. The self-cleaning property of the superwicking Si further holds great promise for addressing the challenges faced in practical seawater desalination or wastewater recycling applications.

### Self-thermal-management of superwicking-FROC

**Fig. 4:**
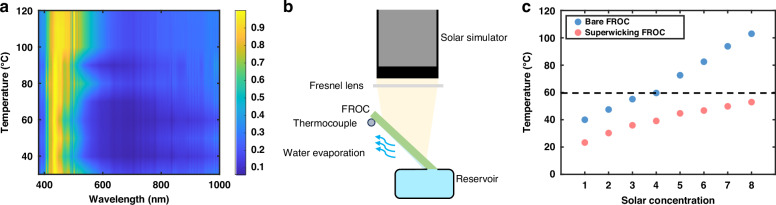
**Evaluation of self-thermal-management for the superwicking-FROC. a** Reflectance of the as-deposit FROC measured at 45° incidence under different temperatures. Negligible variation can be seen for temperatures under 70 °C, while spectrum broadening and red shifting can be observed at higher temperatures. **b** Schematics of evaluating the self-thermal-management function for the superwicking-FROC. **c** Comparison of the steady state temperature reached for the bare FROC and the superwicking-FROC under different solar concentrations, respectively

The thermal stability of FROC is of critical importance, i.e., the spectral response ideally should have little variance even when working at an elevated temperature. To characterize FROC’s thermal performance, we test FROCs at different temperatures by mounting it under a solar simulator at 45°. The reflected light is then collected using a 10x objective lens and directed by fiber collector towards a spectrometer (Photon Control: SPM001, wavelength range: 300–1000 nm). Figure 4(a) shows the measured normalized reflectance of FROC over a temperature range from 30 °C to 120 °C at an interval of 10 °C. Here, the spectra collected from the solar simulator are used as references for normalization. At lower temperatures (e.g., < 70 °C), the spectral response of the FROC remains nearly unchanged. However, when temperature increases, we observe a broadening of the reflection band and a red shift of the resonant wavelength at the reflection peak. This is most likely due to the thermal expansion of the coating layers and may lead to a reduction in performance and heat accumulation in the PV cell.

When the FROC is turned superwicking, continuous water evaporation is established at the backside of the substrate. Not only will this allow highly efficient water desalination by utilizing the heat stored within the FROC, but also realize a self-thermal-management functionality to keep the temperature of the FROC at a low level through the evaporative cooling effect. In such a way, the broadening and red shifting of the PV band occurred at high temperature can be effectively avoided. Following the optimal laser pulse energy, we turn the backside of the as-deposit FROC superwicking, with an active area of 30 mm × 70 mm. Figure 4(b) shows the experimental setup for testing the self-thermal-management functionality of the superwicking-FROC. The superwicking-FROC is positioned at 45° with the bottom just touching the water surface within the reservoir filled with 3.5% wt saline water. The light intensities from the solar simulator are varied such that the solar concentration gradually increases. Once a constant evaporation process is established and thermal equilibrium is reached for each light intensity, the temperature of the superwicking-FROC is recorded and compared to the bare FROC, as shown in Fig. 4(c). For all the solar concentrations used up to 8, the temperature of the superwicking-FROC is constantly regulated below 60 °C. Therefore, superwicking-FROC provides effective thermal management to ensure stable optical response even under strong solar concentrations.

### Characterization of the superwicking-FROC HPT system

**Fig. 5:**
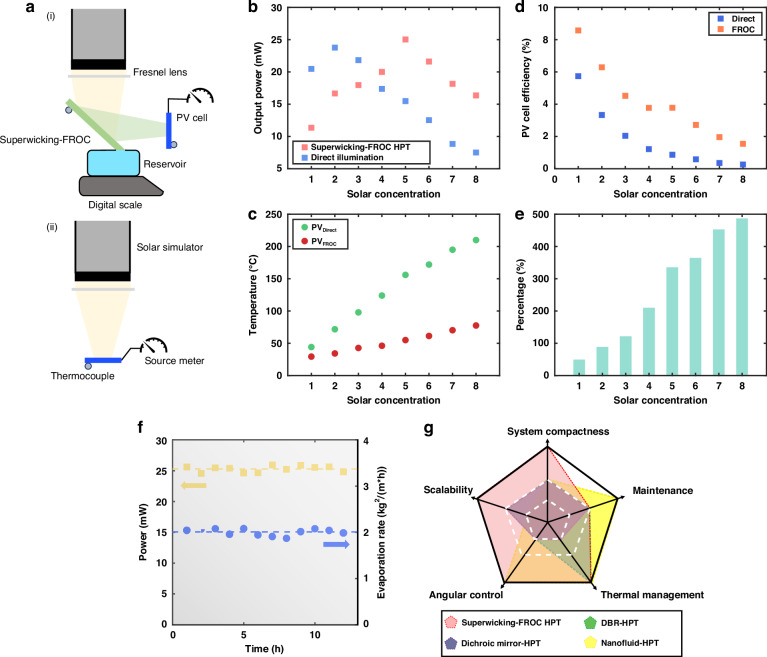
**Characterization of superwicking-FROC HPT system. a** Schematics of the (i) superwicking-FROC HPT system and (ii) directly illuminated PV cell, respectively. **b** Generated electricity of the PV cell under different solar concentrations for the superwicking-FROC HPT system and the directly illuminated PV cell, respectively. **c** Comparison of temperature for the PV cell in the two systems, respectively. The temperature of the PV cell with direct illumination is always higher, and the temperature difference rapidly increases as the concentration becomes larger. **d** Comparison of PV cell conversion efficiency for the superwicking-FROC HPT system and direct illumination, respectively. **e** Corresponding PV cell operating efficiency increase for the superwicking-FROC HPT system compared to direct illumination. **f** Generated electricity from the PV cell (yellow squares) and evaporation rate from the superwicking Si (blue circles) throughout the 12 h measuring period. The average values are marked with dotted lines. **g** Comparison between our superwicking-FROC HPT system vs. other thermally decoupled HPT platforms (DBR-HPT^16^, dichroic mirror-HPT^43^, and Nanofluid-HPT^44^)

Following the examination of the self-thermal-management functionality of the superwicking-FROC, a compact superwicking-FROC HPT system is built in conjunction with a 20 mm × 20 mm truncated single-junction amorphous Si PV cell, as schematically shown in Fig. 5(a, i). The front side of the superwicking-FROC effectively reflects the desired PV band from the concentrated solar to the amorphous Si PV cell for electricity generation and simultaneously absorbs the rest solar energy as heat with minimal thermal losses. Meanwhile, the back side utilizes the thermal energy for efficient water desalination while maintaining a low temperature to keep constant optical responses through self-thermal management. The superwicking-FROC is positioned at 45° to partially reflect the concentrated solar light to the vertically mounted PV cell, where both the FROC and the PV cell are thermally insulated to prevent unintended heat exchange with the mounting frames. Meanwhile, the bottom of the superwicking-FROC is in contact with 3.5% wt saline water in the reservoir for water desalination.

As a concrete comparison, a directly illuminated PV cell (Fig. 5(a,ii)) with the same surface area is used as a reference to represent a standalone solar-PV system. Thermocouples are affixed to the backside of both the superwicking-FROC and the PV cell to record the respective temperature (see Methods). Figure 5(b) presents the PV cell’s output power under direct illumination and within superwicking-FROC HPT system with varying solar concentrations (${C}_{{opt}}$) from 1 sun to 8 suns, respectively. Under direct illumination, the output power of the PV cell initially increases but rapidly declines beyond ${C}_{{opt}}$ > 2. At lower ${C}_{{opt}}$, the energy absorption corresponding to the bandgap energy outweighs the efficiency deterioration induced by heat accumulation and resulting in power increase. However, as ${C}_{{opt}}$ becomes larger, the heat rapidly accumulates, and the temperature of the PV cell exhibits a near-linear increase, reaching above 200 °C at ${C}_{{opt}}$ = 8, as shown in Fig. 5(c). At such high temperature, the performance of the PV cell drops significantly, resulting in a mere 6 mW output power at ${C}_{{opt}}$ = 8. For the superwicking-FROC HPT system, however, the thermal accumulation on the PV cell is substantially mitigated, as the highest temperature recorded at ${C}_{{opt}}$ = 8 being only 77.6 °C. Consequently, the output power of the PV cell continues to increase with ${C}_{{opt}}$, reaching a maximum of 25.03 mW at ${C}_{{opt}}$ = 5 (Fig. 5(b)). At this solar concentration, the PV cell shows a 101 °C lower temperature compared to the standalone solar-PV system (Fig. 5(c)), implying a potential twenty-fold extended lifetime of the PV cell^4^.

One crucial performance metric for PV cells is the conversion efficiency ${\eta }_{s-e}={P}_{e}/{P}_{i}$, where ${P}_{e}$ and ${P}_{i}$ denote the generated electrical power and the total incoming power, respectively. The conversion efficiency of the amorphous Si PV cell employed in our measurements is summarized in Fig. 5(d). As expected, ${\eta }_{s-e}$ decreases with increasing ${C}_{{opt}}$ for both cases, indicating the performance degradation resulting from heat accumulation within the PV cell. However, PV cells from the superwicking-FROC HPT system consistently exhibit higher conversion efficiency compared to the same PV cell operating with direct solar illumination. At ${C}_{{opt}}=5$, where the PV cell has the highest output power, the use of superwicking-FROC leads to a remarkable enhancement in ${\eta }_{s-e}$ by 335.7%, as shown in Fig. 5(e).

Under the solar concentration ${C}_{{opt}}=5$, we conducted a 12 h measurement for the superwicking-FROC HPT system (relative humidity 40%). Over the measuring period, the system maintains an average output electricity of 25.3 mW and water evaporation rate of 2 ${kg}{m}^{-2}{h}^{-1}$ (Fig. 5(f)), with minor fluctuations likely due to solar simulator power instabilities. The temperatures of both the PV cell and superwicking FROC remain stable during the measurement, with respective average temperatures being 55 °C and 46 °C (Fig. S5(a)). Note that the PV temperature is higher than the FROC only because the FROC is conducting water desalination at the back side. The bare FROC always has a higher steady-state temperature than the PV cell under the same solar concentration. Our numerical calculations also reveal similar results by considering the power conservation within the superwicking-FROC HPT system (Fig. S5(b)).

The evaporation performance is quantitatively determined by the evaporation efficiency, denoted as ${\eta }_{{evap}}=\frac{\dot{m}{A}_{{SW}}{H}_{{LV}}}{A{C}_{{opt}}\int I\left(\lambda \right)\alpha \left(\lambda \right)d\lambda }$. Here, $\dot{m}$ is the evaporation rate ${H}_{{LV}}$ represents the latent heat of water evaporation, ${C}_{{opt}}$ is the optical concentration, $I\left(\lambda \right)$ corresponds to the 1.5AM solar irradiance, $\alpha \left(\lambda \right)$ signifies the absorption of the superwicking FROC and *A*_*SW*_ and *A* are the respective surface area of the evaporation region and the concentrated solar at the PV cell plane. From our measurements, superwicking-FROC HPT system achieves an average evaporation efficiency of ${\eta }_{{evap}}=57.3 \%$. Moreover, the temperature of the bulk water in the reservoir is increased by ~2 °C during the measurement, implying additional thermal storage capability of the superwicking-FROC HPT system through the conductive heat transfer from the superwicking-FROC to the bulk water (Fig. S5(c)). Combining with the average solar-photovoltaic conversion efficiency from the PV cell, we have realized an efficient superwicking-FROC HPT system with an overall working efficiency of 79.6%. Moreover, the majority of the active evaporating area remains free from salt accumulation during the measurement (Fig. S5(d)). By measuring the mass of the collected salt, a ~ 99.36% salt-extracting efficiency is achieved (Fig. S5(e)). These results underscore the stability of the superwicking-FROC HPT system, affirming its potential for sustainable and efficient solar energy harnessing for simultaneous electricity generation and on-site water desalination.

## Discussion

In this study, we present a compact and novel configuration of a superwicking-FROC HPT system. This hybrid system is cost-effective, low iridescent, energy-efficient, and highly compact, which can effectively separate the photovoltaic (PV) spectrum while directly harnessing the thermal component from sunlight. Our approach involves the utilization of a specially designed superwicking-FROC that serves the dual purposes as a quad-band spectral filter as well as an interfacial evaporator. The simple four-layer coating on the front side allows us to achieve the reflection of a narrow PV band to the amorphous Si PV cell, subsequently mitigating the heat accumulation and storing the excess heat within the substrate from the two thermal bands with minimal radiative heat loss. This enables the single-junction PV cell to work beyond AM 1.5 G solar illumination while maintaining a proper working temperature. Meanwhile, single-step femtosecond laser writing is employed to turn the backside of the substrate superwicking, allowing on-site interfacial desalination as well as self-thermal-management to maintain the spectral response. The superwicking-FROC HPT system consistently exhibits superior performance, with a sustained high evaporation rate of 2 ${kg}{m}^{-2}{h}^{-1}$ and steady photoelectric conversion, yielding 25.3 mW of output power from the amorphous Si PV cell over a 12 h testing period. This represents an overall conversion efficiency of 79.6% for the superwicking-FROC HPT system. Note that the interfacial solar desalination can also be used to directly cool a solar cell in a thermally coupled HPT system, which will be explored in our future studies. Figure 5(g) compares the performance metrics of superwicking-FROC HPT system reported in this work with other thermally decoupled HPT platforms. Our system outperforms existing platforms by providing low-cost scalability, ultra-compactness, excellent thermal management, and flexible angular control. Because thin film structures are prone to environmental degradation, they require additional protective covers and more maintenance compared to nanofluid-HPT. To enhance durability, ceramic materials could be used to make FROCs^41^. The advancement in this study offers a scalable, cost-effective solution poised for widespread adoption of HPT systems, potentially transforming the renewable energy and water purification markets.

## Materials and methods

### Deposition of FROC

The FROC was deposited on a P-type silicon substrate using an electron beam evaporation (EBE) system (Kurt J. Lesker PVD 75) under a vacuum of 5 × 10^−6^ Torr, where the deposition rate for Ge, Ni, TiO_2_, and Ag were 0.1 $\mathring{\rm A} {{s}}^{-1}$, 0.1 $\mathring{\rm A} {{s}}^{-1}$, 0.5 $\mathring{\rm A} {{s}}^{-1}$, and 0.5 $\mathring{\rm A} {{s}}^{-1}$, respectively.

### Numerical calculation of the reflection and absorption spectra

Numerical reflection and absorption spectra were calculated using the transfer matrix method (TMM) written in MATLAB. Absorption is complementary to the calculated reflection and transmission, that is, A = 1 – R – T, and is complementary to only the reflectance for opaque substrates.

### Optical absorbance measurements in the ultraviolet–visible–near-infrared and mid-infrared regions

The hemispherical optical reflectance of the as-deposit FROC was measured in the spectral range of 0.25–2.5 µm using a PerkinElmer Lambda-900 double-beam spectrophotometer coupled with a 50 mm-diameter integrating sphere. Similarly, hemispherical reflectance in the mid-infrared region (2.5–25 µm) was measured using a Thermo Fisher Scientific Nicolet 6700 FTIR spectrometer coupled with a PIKE research integrating sphere. As the FROC is opaque, the absorbance is complimentary to the measured reflectance in ultraviolet–visible–near-infrared and mid-IR range; therefore, absorbance is obtained using A = 1 − R. For the visible reflection at oblique angles, we employed a home-built experimental setup, as shown in Fig. S1(d). Light from a halogen white light source (Thorlabs: SLS301, wavelength range: 360–2700 nm) was first collimated by the combination of an iris and an objective lens, whose polarization was then selected by a broadband Glan–Thompson polarizer (Melles Griot: 03 PTH 112/C, wavelength range: 350–2300 nm). Eventually, the reflected beam from the FROC went through another broadband Glan–Thompson polarizer and was coupled into the fiber collector of the spectrometer (Photon Control: SPM001, wavelength range: 300–1000 nm) by an objective lens. The measured reflectance of the FROC was normalized to the reflectance of an Ag mirror. For reflection at oblique angles of the FROC in the spectral range of 2.5–25 µm, a PIKE 10Spec accessory was used to measure the reflection spectra at incident angle of 10°, while a PIKE VeeMAX III was used to measure the reflection spectra at incident angle of 30 – 5 – 80°.

### Photovoltaic measurements

A solar simulator (Sanyou) with an AM1.5 G airmass filter was first calibrated for 1 Sun (1000 W m^−2^) using an NREL-certified PV reference solar cell. The readings from a thermopile power meter (FieldMax II TO, Coherent; minimum measurable power ±10 μW) with the working wavelength adjusted to 500 nm is used as a unit of one-sun concentration. The head of the power meter is circular with a diameter of 20 mm. Therefore, a reading of ~314 mW from the power meter corresponds to 1000 W/m^2^ incident flux from the solar simulator. A Fresnel lens with a 250 mm focal length was mounted at the output port of the solar simulator to vary the optical concentration. The current in the xenon lamp was varied to adjust solar irradiance from 1000 W m^−2^ (314 mW from the power meter reading) to 8000 W m^−2^ (2512 mW from the power meter reading) at the plane of the PV cell. Every time the current was changed, we waited for 20–30 min for the solar simulator to stabilize. Then, the power meter was placed at the PV cell plane for 5–10 min to obtain stabilized readings. The amorphous Si PV cell was purchased, cut, and two wires were soldered to create a functioning PV cell. The temperature was measured using thermocouples, and we reported the equilibrium temperature. Power-voltage curves were obtained using a Keithley 2400 source meter by sweeping the voltage in a certain range, and maximum powers were found at the peak of the curve. All the optical concentrations mentioned in the manuscript are given in terms of the truncated amorphous Si PV cell under direct illumination.

### Sample fabrication of superwicking Si

A Ti:sapphire Astrella ultrafast laser from Coherent with a center wavelength of 800 nm, pulse duration of 50 fs, and repetition rate of 1 kHz was used for the fabrication. The output beam was delivered to a Galvano scanner through a series of optics to realize line scanning on the back side of a P-type Si wafer with 0.5 mm thickness. The sample was mounted on an *z* translational stage with a resolution of 4 μm with the backside facing towards the incoming laser beam and was scanned normal to the fs laser beam. In a typical experimental procedure, the laser beam was focused onto the target surface through the Galvano scanner with a focal spot size of ∼40 µm using a planoconvex lens with a focal length of 100 mm and scanned in a spiral manner. For different samples, the pulse energy is controlled by a neutral density filter placed in the optical path. A scanning periodicity of 100 μm was used for all samples to avoid overlapping of adjacent scanning path and minimize the untreated area as well. For industrial applications, ultrafast lasers with higher power and faster repetition rates can be used, which can significantly reduce the fabrication time and cost^42^.

### Surface topography and surface morphology measurements

The surface topography and depth profile of microgrooves on the superwicking Si were measured using a 3D confocal laser microscope (Keyence VK 9710-K) with an elevation resolution of 0.2 µm. The surface morphology of the superwicking Si surface was measured using a SEM/FIB Zeiss-Auriga scanning electron microscope at different magnifications to visualize the micro- and nano- structures inside the microgrooves.

### Wetting dynamics measurements

The wetting dynamics of the superwicking Si were measured by mounting the sample onto a vertical platform, and 200 µL water drop was delivered at the bottom of the sample by a pipette. The wetting dynamics were then captured by a digital camera at 30 frames per second. The recorded videos were then loaded onto our in-house MATLAB analysis code to generate Fig. S3(a).

### Evaporation measurements for characterizing superwicking Si

The evaporation performances of the superwicking Si presented in Fig. 4(e) were measured by a digital scale with an accuracy of 0.001 g (Navada Weighing SMB-60). The experimental conditions were maintained at a temperature of 20 °C and a relative humidity of 40% for all measurements by putting the setup in an HVAC-controlled lab. A 5 × 5 × 5 cm water reservoir is used to supply distilled water to the superwicking Si during the evaporation measurement, where a 1 × 10 mm slit was drilled by fs laser to insert the sample. For evaporation performances under different ambient temperatures, a home-built environmental chamber made from insulating foams was used. To maintain the chamber temperature at a certain level, a 100 W heater with PID controller was placed inside the chamber, with which the temperature inside the chamber can be controlled within ±1 °C. For each ambient temperature measurement, the setup was first placed inside the chamber for 15–20 min until a stable ambient temperature has been reached and continuous evaporation has been established.

## Supplementary information

Supplementary Information for Hybrid Solar Photovoltaic Conversion and Water Desalination via Quad-band Fano-Resonant Optical Coatings and Superwicking Cooling

## Data Availability

All data needed to evaluate the conclusions in the paper are present in the paper and/or the Supplementary Materials.
