# Supplementary material for: Hybrid solar photovoltaic conversion and water desalination via quad-band fano-resonant optical coatings and superwicking cooling
Source: Light Sci Appl. 2025 Apr 17;14:165. doi: 10.1038/s41377-025-01796-z (PMC12006521; doi:10.1038/s41377-025-01796-z)
Supplement: Supplementary file 1 — Supplementary Information for Hybrid Solar Photovoltaic Conversion and Water Desalination via Quad-band Fano-Resonant Optical Coatings and Superwicking Cooling [file 41377_2025_1796_MOESM1_ESM.pdf]

Supplementary Information for

Hybrid Solar Photovoltaic Conversion and Water Desalination via Quad-band Fano-Resonant Optical Coatings and Superwicking Cooling

Ran Wei[1], Tianshu Xu[1], Mingjiang Ma[1], Mohamed Elkabbash[2,*], and Chunlei Guo[1,*]

[1]The Institute of Optics, University of Rochester, Rochester, NY 14627, USA

[2]Wyant College of Optical Sciences, University of Arizona, Tucson, AZ 85721, USA

* *melkabbash@arizona.edu;chunlei.guo@rochester.edu*

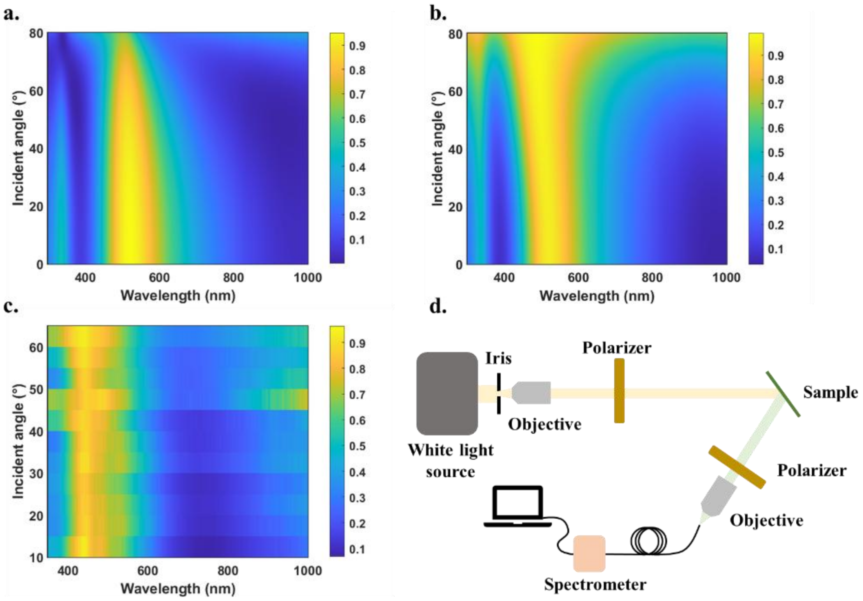

Figure S1 Calculated reflectance of the designed FROC at different angles under **(a)** p-polarized and **(b)** s-polarized illumination. **(c)** Measured angular reflectance of the as-deposit FROC for the s-polarized light. **(d)** Schematic drawing of the experimental setup for measuring the angular response of the as-deposit FROC.

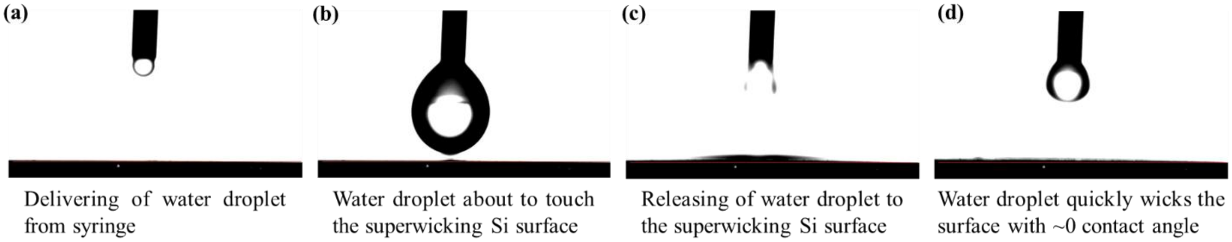

Figure S2 Contact angle (CA) measurement for the superwicking Si surface, where a near-zero CA is achieved by the laser-architected surface.

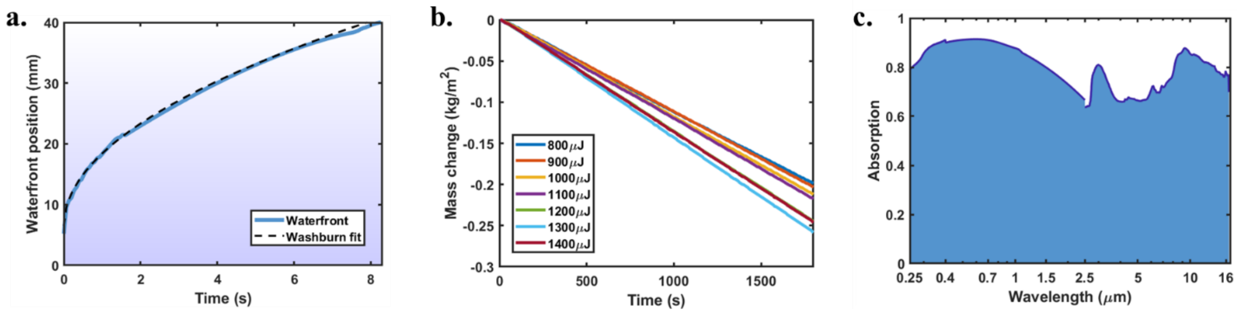

Figure S3 (a) Time-varying waterfront positions for wicking a 40 mm long sample, which finds great agreement with Lucas-Washburn equation. **(b)** Mass change curves for the superwicking Si surface fabricated at different pulse energies. **(c)** Absorption spectrum of the superwicking Si in the solar and IR spectral range.

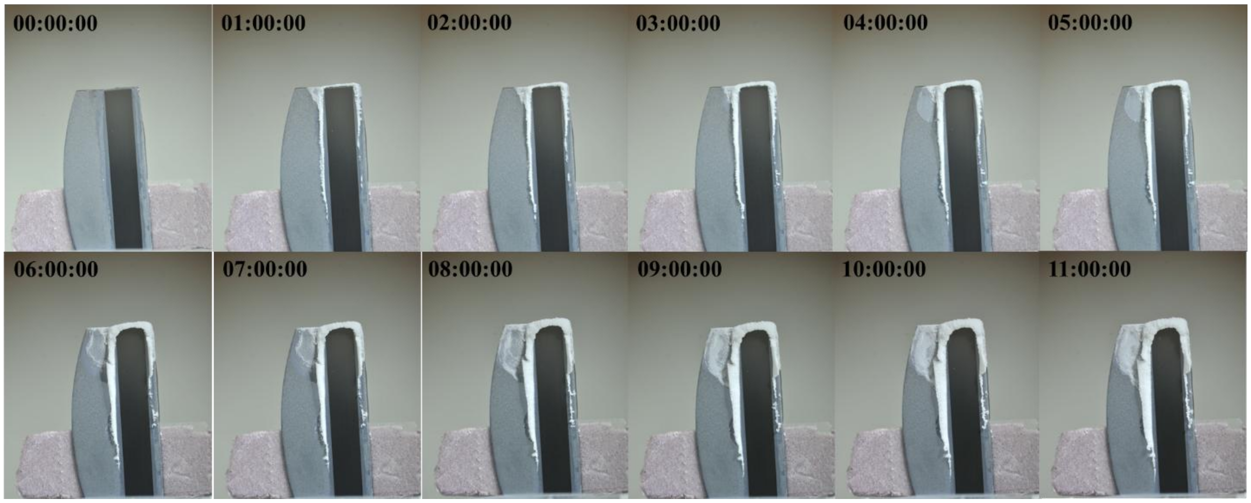

Figure S4 12-hour consecutive self-salt-rejecting testing for the superwicking Si surface at an ambient temperature of 40 ℃. Saline water with concentration of 3.5 % was used as the stimulated seawater. A Nikon D90 camera with an AF-S VR Micro-Nikkor 105 mm camera lens was used to take photos of the surface conditions of the superwicking Si every hour for a consecutive 12-hour period from outside through a 2-inch-diameter optical window. The salt initially crystalized around the edges of the superwicking area and gradually grew outwards, leaving the active evaporation area clean all the time. We believe the self-clean phenomenon is a combined result of coffee-ring [1] and the salt creeping effect [2], where the former one induces initial crystallization of salt at the edge of the active area and the latter precipitates the salt crystallization far from the evaporating salt solution boundary.

Supplementary Note 1 Ideal spectral response of the spectrum splitter in thermally decoupled HPT system and optimization of FROC design

An ideal spectrum splitter in a thermally decoupled HPT system should have the spectral response as shown by the red line in Fig. S5(a). This includes a narrow reflective band with 100 % reflection aligning with the external quantum efficiency of the amorphous silicon solar cell, two thermal bands with 100 % absorption covering the UV and near IR region of the solar spectrum, and an IR band with 100 % reflection to minimize the radiative heat loss. We compare this spectral response with another spectral response that has 100 % absorption in the IR band to see the significance of the low-emissivity IR band, as shown by the green line in Fig. S5(a). This spectral response is shifted vertically by 5 % to allow better visualization.

To evaluate the difference in the solar-thermal performance, we calculate the steady-state temperatures (SSTs) for the two spectrum splitters considering the convective and radiative heat transfer with the ambience as the only thermal loss. The energy conservation holds that $P_{solar} = P_{conv} + P_{rad}$, where $P_{solar} = C_{opt}A_{FROC}\int \alpha_{FROC}(\lambda)I(\lambda)d\lambda$, $P_{conv} = h_a A_{FROC}(T_{FROC} - T_{ambient})$, and $P_{rad} = \varepsilon_{FROC}\sigma A_{FROC}(T_{FROC}^4 - T_{ambient}^4)$. Here, C_{opt} is the solar concentration, A_{FROC} is the area of the FROC, $\alpha_{FROC}(\lambda)$ denotes the absorption of the FROC, $I(\lambda)$ is the AM 1.5 G solar irradiance, h_a represents the free convection coefficient, ε_{FROC} is the emissivity of the FROC, σ signifies the Stefan-Boltzmann constant, and T_{FROC} and $T_{ambient}$ are the SST of FROC and ambient temperature (taken as 22 ℃). Figure S5(b) shows the calculated SSTs for the two spectrum splitters under various solar concentrations. The spectrum splitter with a 100 % reflection in the IR band always has a higher SST than the spectrum splitter with a 100 % absorption in the IR band, implying that the radiative heat loss due to the IR band significantly influences the solar-thermal performance. We further calculate the radiative power loss for the two spectrum splitters, as shown in Fig. S5(c). A significant amount of power is dissipated radiatively when the spectrum splitter has a perfect absorption in the IR region compared to a perfect reflection in the IR region. Therefore, an ideal spectrum splitter should have a spectral response as represented by the red line in Fig. S5(a).

However, designing a spectrum splitter with the exact ideal spectral response is difficult, especially in realizing the sharp transition edges between different bands. In the real case, transition bands should exist between different bands, and our design of FROC optimizes the transition bands in harvesting the most solar energy for the photovoltaic and photothermal subsystems, respectively. Here, we provide the optimization process in designing the proper spectral response of FROC. Five distinct designs incorporating different metal materials in the middle layer are presented, as shown in Figs. S5(d). For each design, the thickness for each layer in FROC is varied. As a result, these designs consist of Ge (10 nm) – Ag (20 nm) – TiO_2 (63 nm) – Ag (100 nm), Ge (10 nm) – Cu (15 nm) – TiO_2 (68 nm) – Ag (100 nm), Ge (9 nm) – Ni (9 nm) – TiO_2 (73 nm) – Ag (100 nm), Ge (10 nm) – W (10 nm) – TiO_2 (73 nm) – Ag (100 nm), and Ge (8 nm) – Pt (8 nm) – TiO_2 (73 nm) – Ag (100 nm). All designs have the same reflection peak at ~ 500 nm, corresponding to the peak of the external quantum efficiency of the amorphous silicon solar cell.

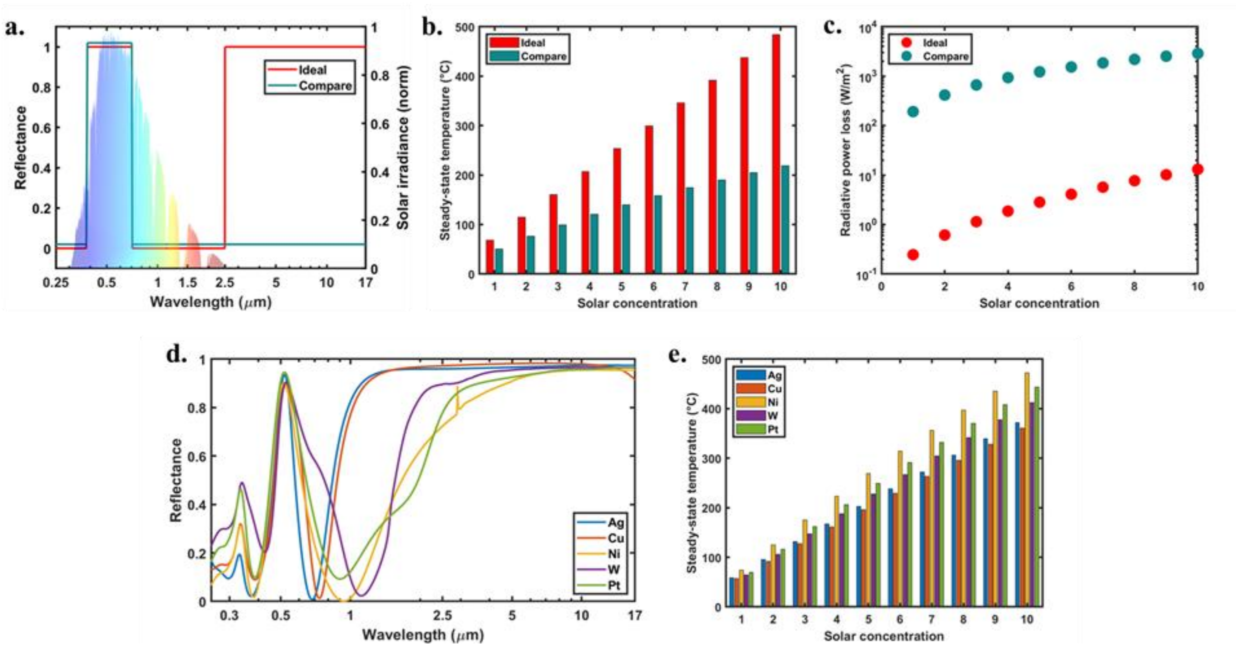

Figure S5: (a) Spectral responses for an ideal spectrum splitter and a spectrum splitter with 100% absorption in the IR band. **(b)** Calculated SSTs for the two spectral responses under various solar concentrations. **(c)** Calculated radiative power loss for the two spectral responses under various solar concentrations. **(d)** Spectral responses for five different FROC designs incorporating different metal materials. **(e)** Calculated steady-state temperatures for the five FROCs under various solar concentrations.

To find out which FROC design has the best solar-thermal performance, we calculate their SSTs following the same process as before. The calculated SSTs for different FROC designs under various solar concentrations are summarized in Fig. S5(e), where the Ni-based

FROC has the highest SST among all five designs. Moreover, the SSTs of the Ni-based FROC are also close to the SSTs of the spectrum splitter with the ideal spectral response. This proves that the FROC design used in the manuscript can harvest the most solar energy through the thermal bands and its spectral response is closest to the ideal spectral response.

Supplementary Note 2 Verification of evaporation rate of the superwicking Si with elevated temperature

To validate our observation in Fig. 3(e), we recorded the superwicking Si surface temperature using a k-type thermocouple when constant evaporation is established and the relative humidity in the environmental chamber with a humidity sensor under different ambient temperatures. Accordingly, the saturation vapor pressure at the surface of the superwicking Si surface and the partial vapor pressure in the air can be obtained from the psychrometric chart [3]. These data are summarized in Table S1 as shown below.

Table S1: Summary of measurement data under different ambient temperatures.

Ambient temperature (℃)	Relative humidity (%)	Partial vapor pressure (Pa)	Surface temperature of the superwicking silicon (℃)	Saturation vapor pressure (Pa)	Evaporation rate ($kgm^{-2}h^{-1}$)	r
25	40	1354.56	23.6	2878.43	0.72	2116
30	33	1388.42	26.4	3352.52	0.91	2158
35	24	1371.49	29.1	3962.08	1.21	2140
40	18	1371.49	33.3	5147.31	1.78	2110

The evaporation rate of the superwicking Si surface is proportional to the difference between the saturation vapor pressure at the evaporating surface and the partial vapor pressure in the ambient air. Therefore, $r = (P_{sat} - P_{partial})/\dot{m}$, where P_{sat}, $P_{partial}$, and $\dot{m}$ are the saturation vapor pressure at the evaporating surface, the partial vapor pressure in the ambient air, and the evaporation rate, respectively, should remain a constant for all ambient temperatures. This ratio, r, is expected to remain constant across all ambient temperatures. The calculated values of r are summarized in the last column of Table S1, where we observed similar values across different ambient temperatures. This finding supports our observation of the increase of evaporation rate for the superwicking Si with temperature presented in Fig. 3(e) in the manuscript.

Supplementary Note 3 Numerical calculation of theoretical temperature of FROC, salt rejection for 12-hour testing, and more long-term testing data

By considering the power conservation ($P_{solar} = P_{Rad} + P_{Conv} + P_{Cond} + P_{Evap}$, where the terms represent solar, radiation, convection, conduction, and evaporation energy, respectively) of the superwicking-FROC HPT system, we conducted numerical calculations to determine the theoretical temperature of the FROC required to support the experimental evaporation results presented in Fig. 5(g). The absorbed power by the FROC, denoted as P_{solar}, was calculated as $P_{solar} = A_s C_{opt} \int I(\lambda)\alpha(\lambda)d\lambda$. The dissipation of this incoming power occurs through radiation ($P_{Rad} = \varepsilon A\sigma(T^4 - T_{ambient}^4)$), convection ($P_{Conv} = hA(T - T_{ambient})$), conduction ($P_{Cond} = (T - T_{water})/R_c$), and evaporation ($P_{Evap} = \dot{m}A_{SW}H_{LV}$). Here, T, $T_{ambient}$, and T_{water} are the temperature of the FROC, the ambient temperature, and the bulk water temperature in the reservoir, respectively, $\alpha(\lambda)$ is the absorption of FROC, ε is the emissivity of the FROC, σ is Boltzmann constant, h is the free convection coefficient of air, R_c is thermal resistance for the conductive heat transfer from bulk water to the evaporator surface, $\dot{m}$ is the evaporation rate, and A_s, A and A_{SW} are the concentrated solar area, total area of both the front and back side of FROC, and superwicking area, respectively.

The real-time temperature of both the superwicking-FROC and PV cell during the 12-hour indoor testing can be seen in Fig. S6(a), where the average temperature of superwicking-FROC was further compared to our numerically calculated result in Fig. S6(b). Notably, we found that the theoretical temperature of the superwicking-FROC aligns well with the experimentally measured value. Additionally, in the case for bare FROC where there is no evaporation (i.e., $P_{Cond} = P_{Evap} = 0$, as is the case in Fig. 4(c)), we also observed good agreement between the experimentally measured temperature and the theoretically calculated one of the bare FROC. These findings underscore the accuracy of our numerical calculations and further validate the self-thermal-management performance of the superwicking-FROC HPT system.

Based on the temperature measurement of the FROC, we further calculated the power distribution of the solar-heated FROC, as shown in Fig. S6(c). Most of the absorbed solar power is used to support the fast water evaporation at the superwicking back side, part of the solar power is stored in the water reservoir through the conductive heat transfer from the superwicking-FROC to the bulk water, and the rest of the solar power is dissipated through convective and radiative heat exchange with the ambience. Because of our spectral design, the radiative heat loss is largely suppressed. The heat dissipation may be further reduced by having a transparent thermal insulation material as a topping layer on the FROC, e.g., a silica aerogel [4].

Over the 12-hour testing cycle, the superwicking-FROC HPT system continuously desalinated the saline water with constant high evaporation rate of ~2 $kgm^{-2}h^{-1}$, while the active evaporation area, i.e., the laser-treated area, remained free from salt crystallization. Figure S6(d) shows the surface appearance of the back side of the superwicking-FROC after 12-hour operation, where all salt crystallization occurred on the untreated area and the active area remained nearly clean. The salt was further collected and its mass was measured by a digital scale (Navada Weighing SMB-60 with an accuracy of 0.001g), as shown in Fig. S6(e). The salt extracting efficiency is given by,

$$\eta_{extract} = \frac{m_{salt}}{\dot{m}Atc_{saline}} \qquad (S1)$$

where m_{salt} is the measured salt mass, $\dot{m}$ is the evaporation rate of the superwicking-FROC HPT system, A is the evaporation area, t is the measuring time, and c_{saline} is the concentration of the saline water (3.5 %).

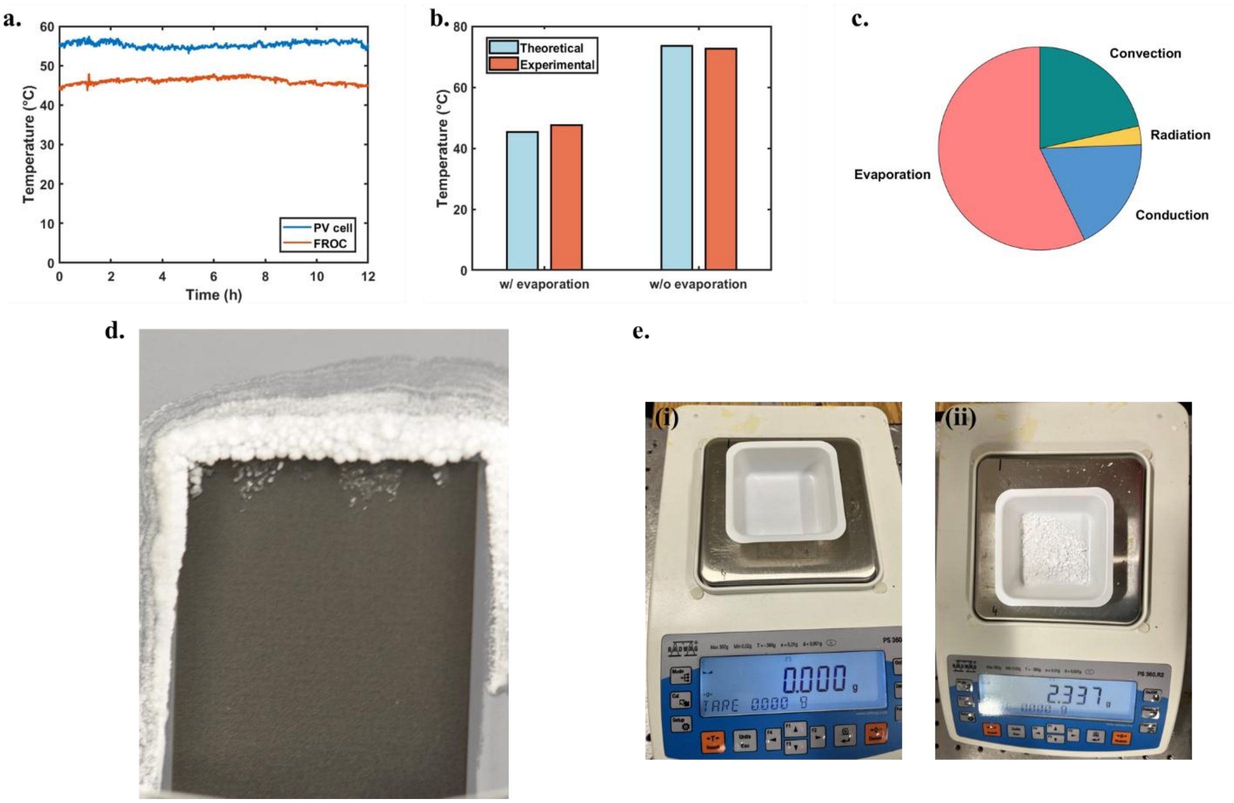

Figure S6 (a) Experimentally measured temperatures for the FROC and PV cell respectively during the 12-hour indoor testing. **(b)** Comparison of the experimentally measured FROC temperature with our numerically calculated value. **(c)** Power distribution in the superwicking-FROC HPT system. **(d)** Photo of the surface appearance of the back of the superwicking FROC after 12-hour operation in desalinating 3.5 %wt saline water. **(e)** Mass of the collected salt from the superwicking FROC surface.

Additionally, the superwicking-FROC HPT system was tested continuously for a week under the solar concentration of 5 to further examine its durability and stability. At the end of each day, the FROC is taken away to measure its spectral response. Figure S7 presents the spectral response of the FROC, the average electricity output, and the average evaporation rate in each day. We find that the system exhibits exceptional stability and durability in providing constant performances for both photovoltaic and photothermal subsystems and maintaining its optical and mechanical integrity as well.

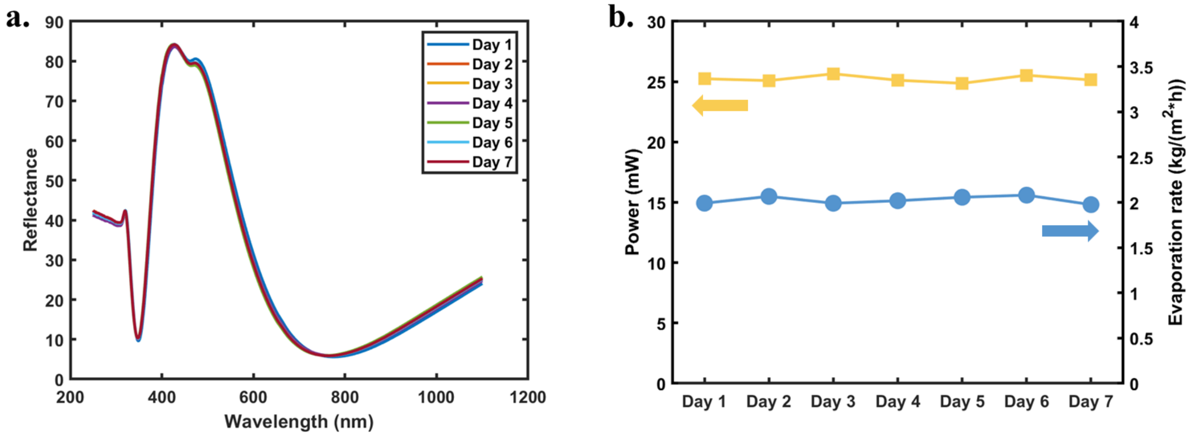

Figure S7: (a) Spectral responses of the as-deposit FROC measured for 7 days. **(b)** Daily average power output from the solar cell and evaporation rate from the superwicking-FROC HPT system.

Supplementary Note 4 Comparison of superwicking-FROC HPT system with other thermally decoupled HPT platforms

Here, we choose three commonly employed thermally decoupled HPT platforms as comparisons with the superwicking-FROC HPT system proposed in this work, i.e., dichroic mirror-HPT, DBR-HPT, and nanofluid-HPT. Dichroic mirror represents the early technologies in thermally decoupled HPT system as a rough splitting technique by separating the spectrum into two parts [5]. Prior to the cut-off wavelength, light is reflected/transmitted to the PV cell, whereas light is transmitted/reflected after the cut-off wavelength to the thermal receiver. DBR is a more advanced splitting technique for realizing better thermal management by reflecting a controlled narrowband towards the PV cell while transmitting the left solar energy to the thermal receiver with minimal thermal loss [6]. This is realized through the stacking of alternating high and low refractive index materials. Nanofluid is synthesized by dispensing nanoparticles (e.g., metallic [7] or dielectric nanoparticles [8,9]) into normal working fluid to selectively absorb the solar energy and transmit the remaining narrowband to PV cell. These three HPT platforms, together with the superwicking-FROC HPT system, are compared in five aspects, scalability, system compactness, maintenance, thermal management, and angular control.

In terms of scalability, FROC only requires a simple four-layer subwavelength thin film coating and thus is cost-effective, while DBR and dichroic mirror require multiple dielectric layers with different refractive indices, adding more fabrication complexity. Nanofluid-based selective absorbers can be stably synthesized in small batches, however, they face problems in large scale production with constant high-quality and homogenous nanoparticles, which require further tolerance evaluation in particle sizes and therefore impact cost of production [10]. For the system compactness, both DBR and Dichroic mirror platforms require additional thermal receivers to complete solar-thermal transition, while nanofluid-HPT necessitates thermal exchanger [11,12]. Conversely, superwicking-FROC HPT system directly uses the heat absorbed by FROC for on-site desalination at the back side, providing the most compact configuration of a thermally decoupled HPT system. When it comes to maintenance, thin-film-based technology (superwicking-FROC HPT system, DBR-HPT, and dichroic mirror-HPT) typically is prone to environmental degradation such as humidity change and contamination. Therefore, they usually require additional protective cover layer and more frequent maintenances than nanofluid-HPT. To enhance durability, ceramic materials can be used to make FROCs [13]. In terms of thermal management (for normal incidence only), dichroic mirror has the worst thermal management among the four platforms since it simply divides the incoming solar energy into two bands. Photon energy larger than the bandgap of the PV cell will be accumulated as heat and therefore degrading the photoelectric conversion performance and lifetime of PV cell. DBR has flexible bandwidth control by tuning the refractive index contrast, thickness, and number of bilayers of the alternating materials. Similarly, nanofluid can also control the bandwidth by strategically distributing nanoparticles with different sizes, effectively absorbing certain rage of sunlight and transmitting a narrowband of sunlight to the PV cell. Therefore, both DBR- and nanofluid-based HPT systems can have similar thermal management performance as superwicking-FROC HPT system. Lastly, DBR and dichroic mirror are all dielectric mirror and have high iridescence, i.e., their spectral responses are highly angle-dependent and have poor angular control [14]. Nanofluid, on the other hand, selectively absorbs the solar energy through effective Rayleigh scattering or localized surface plasmon resonance of the nanoparticles with different sizes suspended in the working fluid, has a comparable angle-insensitive response to the superwicking-FROC HPT system.

Reference

1 Deegan, R. D. *et al.* Capillary flow as the cause of ring stains from dried liquid drops. *Nature* **389**, 827-829 (1997). https://doi.org/10.1038/39827

2 Qazi, M., Salim, H., Doorman, C., Jambon-Puillet, E. & Shahidzadeh, N. Salt creeping as a self-amplifying crystallization process. *Science Advances* **5**, eaax1853 (2019).

3 Szokolay, S. V. Climate analysis based on the psychrometric chart. *International journal of ambient energy* **7**, 171-182 (1986).

4 Zhao, L. *et al.* Harnessing Heat Beyond 200 °C from Unconcentrated Sunlight with Nonevacuated Transparent Aerogels. *ACS Nano* **13**, 7508-7516 (2019). https://doi.org/10.1021/acsnano.9b02976

5 Mitchell, B. *et al.* Four-junction spectral beam-splitting photovoltaic receiver with high optical efficiency. *Progress in Photovoltaics: Research and Applications* **19**, 61-72 (2011).

6 Cao, F. *et al.* Toward a High-Efficient Utilization of Solar Radiation by Quad-Band Solar Spectral Splitting. *Advanced Materials* **28**, 10659-10663 (2016).

7 Crisostomo, F., Becker, J., Mesgari, S., Hjerrild, N. & Taylor, R. A. in *2015 12th International Conference on the European Energy Market (EEM).* 1-5 (IEEE).

8 An, W., Wu, J., Zhu, T. & Zhu, Q. Experimental investigation of a concentrating PV/T collector with Cu9S5 nanofluid spectral splitting filter. *Applied energy* **184**, 197-206 (2016).

9 Huaxu, L. *et al.* Experimental investigation of cost-effective ZnO nanofluid based spectral splitting CPV/T system. *Energy* **194**, 116913 (2020).

10 Mahian, O. *et al.* Recent advances in using nanofluids in renewable energy systems and the environmental implications of their uptake. *Nano Energy* **86**, 106069 (2021).

11 Khullar, V. & Tyagi, H. A study on environmental impact of nanofluid-based concentrating solar water heating system. *International Journal of Environmental Studies* **69**, 220-232 (2012).

12 Ni, J., Li, J., An, W. & Zhu, T. Performance analysis of nanofluid-based spectral splitting PV/T system in combined heating and power application. *Applied Thermal Engineering* **129**, 1160-1170 (2018).

13 Geng, J. *et al.* Wear-resistant surface coloring by ultrathin optical coatings. *PhotoniX* **3** (2022). https://doi.org/10.1186/s43074-022-00061-5

14 Orfanidis, S. J. Electromagnetic waves and antennas. (2002).
